# Conservation of rib skeleton regionalization in the homoplastic evolution of the snake-like body form in squamates

**DOI:** 10.1098/rspb.2024.1160

**Published:** 2024-10-09

**Authors:** Emily J. Hillan, Lucy E. Roberts, Katharine E. Criswell, Jason J. Head

**Affiliations:** ^1^ Department of Zoology, University Museum of Zoology, University of Cambridge, Cambridge, UK; ^2^ Department of Organismal Biology and Anatomy, University of Chicago, Chicago, IL, USA; ^3^ The Natural History Museum, London, UK; ^4^ Department of Biology, Saint Francis University, Loretto, PA, USA

**Keywords:** conservation, ribs, skeletons, regionalization, homoplastic, evolution

## Abstract

Squamates have independently evolved an elongate, limb-reduced body form numerous times. This transition has been proposed to involve either changes to regulatory gene expression or downstream modification of target enhancers to produce a homogeneous, deregionalized axial skeleton. Analysis of vertebral morphology has suggested that regionalization is maintained in snake-like body forms, but morphological variation in the other primary component of the axial skeleton, the dorsal ribs, has not been previously examined. We quantified rib morphology along the anterior–posterior axis in limbed and snake-like squamates to test different regionalization models. We find that the relative position of regional boundaries remains consistent across taxa of differing body types, including in the homoplastic evolution of snake-like body forms. The consistent retention of regional boundaries in this primaxial domain is uncorrelated with more plastic abaxial region markers. Rather than loss of regions, rib shape at the anterior and posterior of the axis converges on those in the middle, resulting in axial regions being distinguishable by allometric shape changes rather than by discrete morphologies. This complexity challenges notions of deregionalization, revealing a nuanced evolutionary history shaped by shared functions.

## 1. Introduction

Although the ‘snake-like’ body form with reduced appendages and an elongated axial skeleton that superficially appears homogenous is widespread across Vertebrata [1–3], the species richness of such taxa in squamates is unparalleled, appearing independently in at least 25 separate lineages [4–6]. The propensity for squamates to evolve elongated limb-reduced forms from limbed ancestors has been the focus of broad evolutionary developmental research [2,7–15]. Previously hypothesized developmental mechanisms driving the transition to a snake-like body form involve deregionalization of the body axis [8], or extreme expansion of the cervical [16,17] or thoracic region [13,18–21]. However, more recent work has demonstrated that the vertebral columns of snakes retain morphological boundaries as seen in limbed squamates [22–27]. Rather than having one homogenous axis, snakes were found to have cryptic regionalization where shape differences between adjacent axial components are subtle and distinguishable only through quantitative methods. While anatomical regionalization of the vertebral column has been investigated, regionalization in the morphology of the other primary component of the axial skeleton, the dorsal ribs, has not. Hence, we applied quantitative morphological analysis to the dorsal ribs of representative squamate species.

Rib morphology is the result of interactions between developmental domains. Like vertebrae, ribs originate from the paraxial mesoderm, referred to as the primaxial domain, and are patterned by *Hox* genes, with regional identity boundaries corresponding to the anterior expression boundaries [18–21,28–31]. Shifts in *Hox* gene expression boundaries are known to result in shifted axial identities for both the vertebrae and ribs [18,32]. However, unlike vertebrae, ribs also interact with the abaxial domain, which gives rise to the lateral plate mesoderm and forms structures including the appendicular and sternal skeleton [33]. The primaxial-derived ribs grow ventrally to invade the adjacent abaxial-derived tissues, pushing the lateral somitic frontier (LSF) [33,34]. While there is clear correspondence between abaxial and primaxial patterns [28], the different tissues have distinct patterning mechanisms as abaxial-derived structures lack the nested, collinear expression of *Hox* genes [21,34,35]. Because the developing ribcage is subject to influences from both abaxial and primaxial domains, examining the patterning of ribcages is key to understanding communication across the LSF. Further, because snakes lack the gross ventral skeletal features seen in limbed taxa, their retained patterning mechanisms are independent of these major abaxial markers, providing a natural dataset for investigating trans-LSF influences.

To test for the presence of regionalization in the dorsal ribcage of squamates, we combined maximum-likelihood model selection with segmented linear regression and geometric morphometrics to model anatomical regionalization of ribs. By sampling representative taxa from every major lineage of squamates of both limbed and snake-like body forms, across multiple independent transitions to the snake-like body form, we determine common features of this transition and infer potential evolutionary mechanisms.

## 2. Material and Methods

### (a) Acquisition of morphological data

We compiled whole-body computerized tomography (CT) scans from adult specimens of 56 species (electronic supplementary material, 1) representing all major squamate clades and *Sphenodon punctatus*. We identify taxa as ‘snake-like’ if they have elongate pre-cloacal skeletons with more than 50 pairs of free dorsal ribs, and girdle reduction or loss (electronic supplementary material, 6). An important exception is amphisbaenian *Bipes canaliculatus*, which bears a uniquely robust pectoral skeleton [36] but is classified as snake-like due to its elongate axial skeleton. We performed data segmentation and rendering using the Thermo Scientific Avizo Software (v. 9.3 lite) and exported ribs from one bilateral half of each specimen as un-smoothed PLY files for landmarking in the Institute for Data Analysis and Visualization (IDAV) Landmark software (v. 3.6). The terminal pre-cloacal, forked rib of snakes was excluded from analysis.

### (b) Three-dimensional geometric morphometrics and segmented linear regression analyses

**Figure 1.**
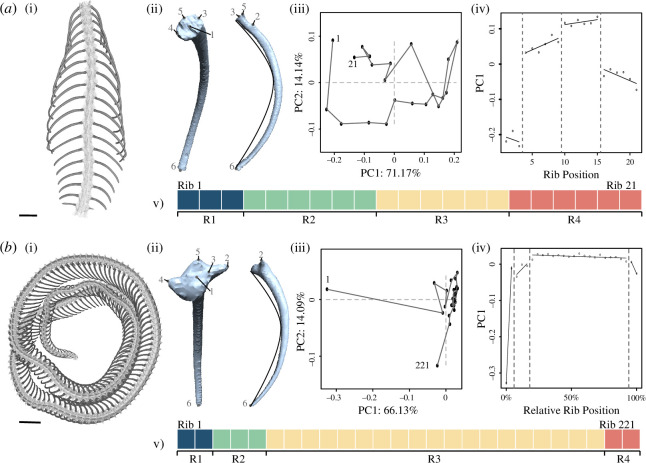
Ribcage regionalization analysis, illustrating the workflow for a representative lizard (*Cordylus cordylus*, (*a*)) and snake (*Pantherophis guttatus*, (*b*)). Analysis begins from (i) the segmented and rendered ribcage, to which (ii) the three-dimensional landmark array (electronic supplementary material, 2) is applied to each rib for limbed taxa or subsampled ribs in elongate species. The landmark scheme is illustrated with points for full landmarks and a curve where the semi-landmark curve would run. The shape variables are extracted, and (iii) PCA is performed. (iv) Segmented linear regression is performed to produce (v) the best-fit regionalization pattern (boxes coloured to indicate statistically distinct regions, coloured sequentially along the axis). R, regions. Scale bars are 5 mm for (*a)* and 20 mm for (*b)*.

We developed a robust three-dimensional landmarking configuration to reliably capture shape data of each rib as an array of three-dimensional Cartesian coordinates (figure 1; electronic supplementary material, 2) including a curve of semi-landmarks along the visceral face of the rib, quantifying curvature [37]. We conducted sensitivity analyses to assess the robustness of the landmark configuration (electronic supplementary material, 3).

We applied this configuration to every rib from every specimen with fewer than 50 ribs and to 26 individual ribs at 4% intervals along the axis for elongate species. This subsampling procedure was performed to account for the effects of parameterization on the regression models for skeletons with different numbers of ribs; it was found to produce repeatable regionalization models with high confidence values (electronic supplementary material, 3). For each species, we consolidated landmark coordinates from each rib to TPS files for input and analysis in R (v. 3.6.3), using packages ‘Morpho’ [38] and ‘regions’ [39]. The code for this and all subsequent analyses is included in full in electronic supplementary material, 4. Procrustes superimposition removed variation in size, orientation and position in the landmark arrays [40] prior to principal component analysis (PCA), producing shape variables. We used segmented linear regression analysis (SLRA) to detect regions and region boundaries along the gradients in shape variables, corresponding to morphological regions that are statistically distinct along the body axis [22]. We used the first six principal components (PCs) for SLRA, accounting for approximately 95% of shape variation per sample, to test for region models of up to six axial regions, allowing for species with increased region numbers. To choose the best maximum-likelihood model, we selected the best model for each of the six regionalization hypotheses, one for each of the possible number of regions, by minimizing the total residual sums of squares. Because models with more regions have more parameters, the likelihood ratios of each could not be directly compared. Model selection would bias towards those with the most parameters, with the same number of segments as there are data points perfectly fitting the data. To account for the bias towards overfitting the data, in addition to the subsampling procedure, we used the modified Akaike information criterion (AIC_c_), which corrects the ratios for the numbers of parameters to allow objective selection of the best-fit model. We accepted best-fit models if the next five top-ranked models were consistent in boundary position. We also recorded the average number of regions, weighted by model probability, as the region score for each taxon. The robustness of this workflow was assessed using sensitivity analysis (electronic supplementary material, 3).

### (c) Analysis of best-fit regionalization models

We calculated the relative axial positions for regional boundaries as the percentage of the total number of ribs (electronic supplementary material, 4–6). We documented abaxial skeletal features including the pectoral girdle and sternal apparatus consisting of the sternum and xiphisternum from original three-dimensional μCT scans to compare the geography of regionalization with potentially associated abaxial structures (electronic supplementary material, 6). The position of the pectoral girdle is defined as the extent of the clavicle and humerus, though the scapula may extend further to the posterior. Positions of vestigial pectoral girdles in snake-like taxa were verified from the literature [36,41–43].

### (d) Rib shape heterogeneity

We calculated the total amount of shape disparity within the rib skeleton in each taxon (axial heterogeneity) as the range of Procrustes distances for all ribs in a sample, following Head & Polly [22]. We tested for significance in shape difference between limbed and snake-like taxa using Procrustes analysis of variance (ANOVA) [44] in the ‘geomorph’ package [45] (electronic supplementary material, 4). To determine the shape difference between rib morphologies at axial extremes versus the mid-point for each taxa, we compare rib shapes 4% and 96% to the mid-point rib by pairwise calculating the shape disparity between Procrustes superposed landmark arrays. To determine the influence of allometric scaling on rib shape, we regressed Procrustes shape variables against the log centroid size, using a Procrustes ANOVA to perform a multivariate regression [44]. Centroid size is used rather than absolute length as the metric size satisfies all criteria for a standard size variable [46] but is uncorrelated with shape in a way that linear measurements could not be [47,48]. Although Procrustes superimposition removes scale, it does not remove allometric variation, which is the shape variation associated with size. By performing this multivariate regression, the resultant residuals represent the component of shape variables independent of their relationship with size [49,50]. We repeated SLRA on these residuals to detect anatomical boundaries that are independent of allometric variables (non-allometric boundaries).

The difference between the region number of the best-fit models using full shape variables versus the non-allometric region number is the number of allometric boundaries.

### (e) Comparative phylogenetics

We constructed time-calibrated phylogenies for sampled taxa using two different topologies based on either molecular [51,52] or morphological [53] data. We obtained first appearance data from the Paleobiology Database (Paleobiodb.org) and TIMETREE [54] (electronic supplementary material, 7, 8). We performed tree construction, first using the ‘paleotree’ package [55] to perform divergence timing estimations with an *a posteriori* dating approach using minimum branch length time-scaling [56], in order to calculate branch lengths. Nodes appear in order of the first appearance of their oldest descendant, according to Smith [57]. Trees were rooted with *Sphenodon punctatus* as an outgroup using the root function from the R package ‘ape’ [58]. Using the package ‘phytools’ [59], we overlaid trait histories for axial heterogeneity on both tree topologies and calculated maximum-likelihood ancestral state reconstructions using fastAnc and mapped them using contMap (electronic supplementary material, 4) to determine nodes on the tree where major shifts in axial heterogeneity occur.

## 3. Results

### (a) Regional boundaries

**Figure 2.**
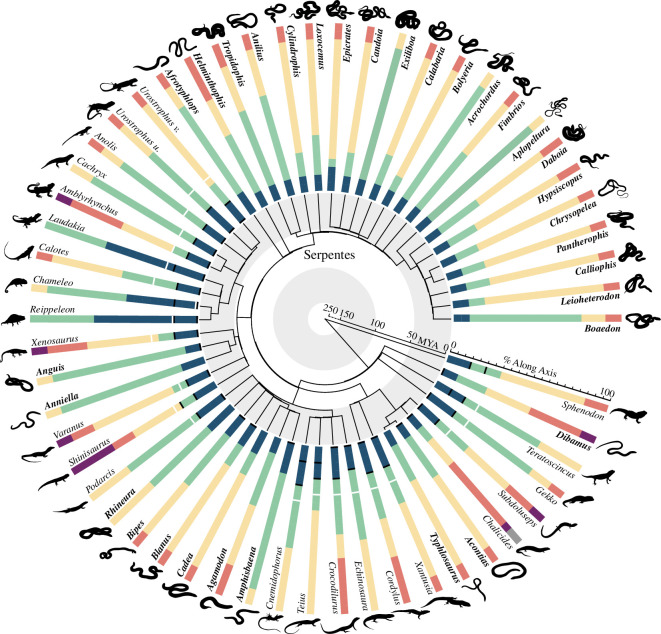
Best-fit models of ribcage regions for each taxon, shown on a radial phylogeny following the molecular-derived topology [51,52]. Snake-like taxa are highlighted in bold. Each ribcage is schematized as a bar with different regions indicated by different colours assigned sequentially along the axis. Black bars indicate the extremities of the pectoral girdle, and white bars indicate the anterior extent of the sternal apparatus, in species where applicable.

**Figure 3.**
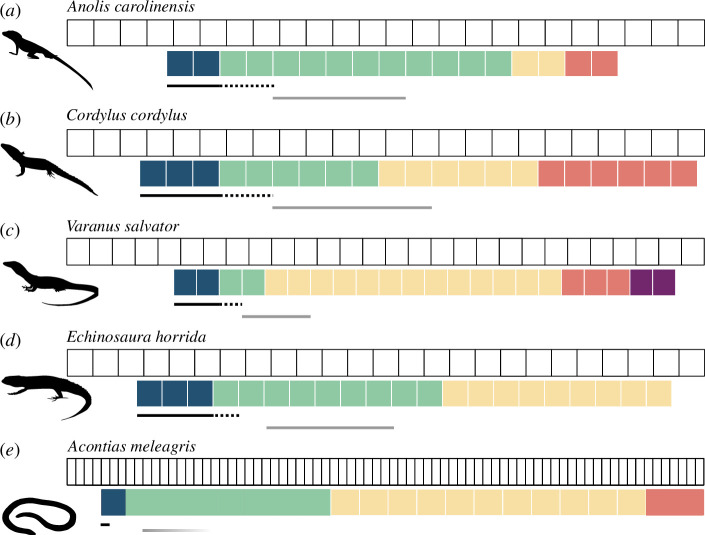
The relationship of ribcage regionalization models to other skeletal features, illustrated for representative taxa: (*a*) the iguanian *Anolis carolinensis*, (*b*) the limbed scincomorph *Cordylus cordylus*, (*c*) the varanid *Varanus salvator*, (*d*) the lacertid *Echinosaura horrida* and (*e*) the snake-like skink *Acontias meleagris*. Best-fit region models are illustrated with colours representing statistically distinct regions along the axis. Coloured boxes represent individual ribs in (*a)*–(*d)*, and bars reflect relative boundary positions in (*e)*. The pre-cloacal vertebral column is shown as white boxes. The positions of the pectoral girdle are indicated by solid black bars, with the dashed black bars showing the extent of the scapulae. Grey bars indicate which ribs articulate with the sternal apparatus, or the anterior extent of parasternal chevrons in the case of *Acontias*.

The majority of taxa have either three or four statistically distinct regions within the dorsal ribcage (figures 2 and 3, electronic supplementary material, 5, 9). Indeed, all snake-like taxa have either three or four regions in their best-fit models (28.13% have three regions and 71.88% have four), while limbed taxa have a more variable number (8.33% have two regions, 25.00% have three, 45.83% have four, 16.67% have five and 4.17% have six). The number of regions is not predicted by body form, as limbed and snake-like forms have no significant difference in region score, with means of 3.96 and 3.81, respectively (*t*(31.01) = 0.71, *p* = 0.49). Across most taxa, we recover a conserved pattern of region boundaries, with an anterior boundary close to the cranium, a posterior boundary relatively close to the posterior extent of the ribcage and one regional boundary in between. This pattern is seen in *Anolis* (figure 3*a*) and with a slight variation in *Cordylus* (figure 3*b*). A deviation from this pattern is exemplified by the best-fit model for *Varanus salvator* having five regions (figure 3*c*).

Clear clade-specific patterns of axial regionalization are present. For example, most acrodontans share elongate anterior regions and reduced region number, seen at the most extreme in *Rieppeleon brevicaudatus* and *Laudakia stellio* each of which only has two distinct regions with a single boundary of 60% along the ribcage from the anterior end in their best-fit models. Conversely, teiids have much longer posterior-most regions related to lumbarization, with an abrupt shortening of ribs in this area, as in *Echinosaura horrida* (figure 3*d*). The variation between members of these clades and related limbed taxa is much higher than the degree of variation seen between snake-like taxa and their closest limbed relatives. For example, the snake-like *Acontias meleagris* shows the conserved four-region pattern (figure 3*e*). Though significant differences are seen between the relative position of the anterior-most boundary in limbed and snake-like forms (17.83% and 6.56%, respectively, *t*(39.72) = 6.93, *p* = 2.45 × 10^−8^) and the posterior-most boundary (77.12% and 88.13%, respectively, *t*(25.48) = −3.22, *p* = 0.004), the differences are in how close the boundaries are to the axial extremes. When comparing limbed and snake-like forms, these boundaries are consistently present and are in similar relative positions.

### (b) Morphological correlates to region boundaries

The position of the anterior-most region boundary is tightly associated with the position of the pectoral girdle in limbed taxa (figures 2, 3 ; electronic supplementary material, 6), with the exceptions of acrodontans that have elongate anterior regions, and in the skink *Chalcides sepsoides,* where the pectoral girdle is anterior to the ribcage. In *Shinisaurus crocodilurus*, there is an additional region break at the anterior of the pectoral girdle. Only 2 of 24 sampled limbed taxa show an association between the position of a region boundary and the anterior rib articulating with the sternal skeleton (figure 2; electronic supplementary material, 6).

The consistent association between pectoral girdle position and anterior region boundary seen in limbed taxa is absent in snake-like taxa. Where pectoral girdles are present, they are positioned at the anterior extreme of the axial skeleton, ranging between the second and fifth vertebra across *Acontias meleagris, Dibamus novaeguineae, Blanus cinereus* and *Amphisbaena fuliginosa*, well anterior to the resolved rib region [36,41,43] (electronic supplementary material, 6; figure 3*e*). In the remaining snake-like taxa and snakes, the girdle has been lost altogether. Similarly, there is no association between the anterior rib region break and the position of the sternal apparatus for snake-like taxa (figure 3*e*). Whereas the amphisbaenian *Bipes* has a full sternum [36], most snake-like squamates only retain parasternal chevrons, unassociated with region boundary position (electronic supplementary material, 6).

**Figure 4.**
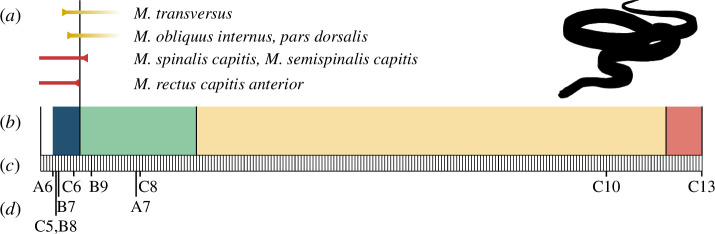
Features associated with rib regionalization in *Pantherophis guttatus*. (*a*) The anterior extents of *M. transversus* (MTr) and *M. obliquus internus*, pars dorsalis (MOID) are marked in yellow, and the posterior extents of the cervicothoracic muscles *M. spinalis capitis* (MSC), *M. semispinalis capitis* (MSsC) and *M. rectus capitis* anterior (MRCA) are marked in red [43]; illustrated in comparison to (*b*) the best-fit model of ribcage regions, with region boundaries illustrated at their relative positions and distinct regions assigned different colours along the axis. Relative positions are shown in comparison to (*c*) the pre-cloacal vertebral column, illustrated as white boxes. (*d*) The position of anterior expression boundaries of *Hox* genes [11,13] is marked.

Non-skeletal features are associated with the anterior rib region boundary of the best-fit model for *Pantherophis guttatus* (figure 4). Axial skeletal positions of muscle origins corresponding to the cervicothoracic boundary, including the posterior origins of primaxial-derived *M. spinalis capitis* (MSC), *M. semispinalis capitis* (MSsC), closely correspond to the model’s region boundary (figure 4*a*) [43]. The anterior expression boundaries of *HoxC6* and *HoxB9*, described for *Pantherophis* in Woltering *et al*. [60] and Di-Poï *et al*. [11], are also found to correspond to within 4% of the anterior-most regional boundary (figure 4*d*).

### (c) Heterogeneity

**Figure 5.**
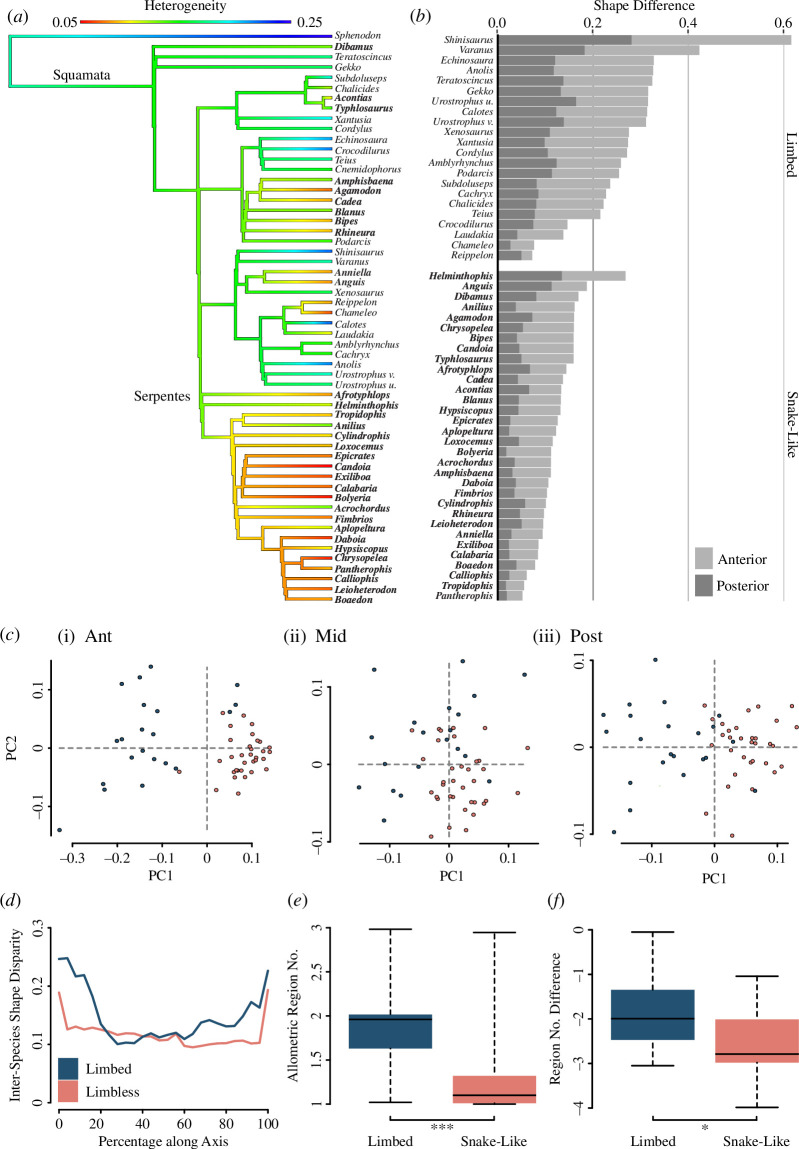
Along-axis shape analysis. (*a*) Intra-axial rib heterogeneity overlaid on the molecular phylogeny of selected taxa with ancestral states for nodes calculated and illustrated. Snake-like taxa are highlighted in bold. (*b*) Shape difference between the mid-axis rib and the anterior rib (light grey) and posterior rib (dark grey) for each species, grouped by body type, in order of total shape difference. (*c*) PCA plots for rib shape sampled at (i) 4%, (ii) 72% and (iii) 96% along the body axis with points representing individual species, coloured by body type (blue for limbed taxa, orange for snake-like taxa). (*d*) Inter-species shape disparity at each axial interval, by body type. (*e*) Boxplot of number of allometric region boundaries by body type. (*f*) Boxplot of the decrease in region score when allometric boundaries are removed, according to body type (region score: allometric region score). * denotes p<0.05, *** p<0.001. Limb-reduced species are highlighted in bold.

Trends across the phylogeny include independent instances of decreasing heterogeneity (figure 5*a*). While values are lower in all squamate taxa than in *Sphenodon punctatus*, ancestral state reconstruction shows that there is a further decrease at the base of each independent lineage with the evolution of the snake-like form (figure 5*a*). Ancestral state reconstruction predicts that at the base of Serpentes, the last common ancestor had a low heterogeneity of 0.11 (95% CI = 0.083–0.14), within the range of extant snakes sampled. Boid and colubrid snakes in particular have low heterogeneity. Heterogeneity is significantly different between snake-like forms (mean = 0.09) and limbed forms (mean = 0.16), (*t*(31.42) = 7.70, *p* = 1.01 × 10^-8^).

Decreased heterogeneity is the result of decreased differences in rib shape along the axis. Snakes and snake-like taxa show a unique pattern of shape change in which changes in rib shape are greater at the ends of the ribcage than within the middle part of the cage. In limbed taxa, there is a greater Procrustes distance between the mid-point rib shape and the shape of ribs at both the anterior (*t*(5.4217) = 26.005, *p* = 1.107 × 10^−5^) and posterior of the body axis (*t*(5.3721) = 27.917, *p* = 1.013 × 10^−5^) (figure 5*b*). Rib shape is most similar between limbed and snake-like taxa at the mid-axial portion, with no significant difference in shape from 50% to 70% along the axis, as determined by Procrustes ANOVA of shape variables for all species at each interval. At the anterior (figure 5*c*(i)) and posterior (figure 5*c*(iii)) axes, the shape variables for limbed or snake-like species cluster distinctly in PCA space, while in the mid-axis (figure 5*c*(ii)), the clusters of shape variables for the different body types overlap with each other. Thus, in snake-like taxa, the shape of ribs in anterior and posterior regions converges towards a mid-axis morphology common to all body forms. This results in lowered between-species variation for anterior and posterior regions in snake-like taxa (figure 5*d*).

By having the anterior and posterior rib morphology more similar to the mid-axis shape (figure 5*b*), region boundaries in snake-like taxa are less representative of size-independent pure-shape but are scaling-related changes. All limbed taxa have at least one non-allometric boundary, with exceptions for iguanians. In all but four snakes, there are no non-allometric boundaries, with only *Helminthophis frontalis, Bolyeria multocarinata, Fimbrios klossi* and *Calliophis maculiceps,* showing middle boundaries defined by shape change independent of association with size. Comparing limbed and snake-like taxa, despite there being no significant difference in region score (*t* = 0.350, d.f. = 30.996, *p* = 0.729, mean in group limbed (3.865), mean in group snake-like (3.788)), there is a clear and significant difference in the region score when allometric boundaries are removed (*t* = 4.399, d.f. = 41.618, *p* = 7.399 × 10^−5^, limbed mean = 1.880, snake-like mean = 1.281) (figure 5*d*). The relationship between body type and this decrease in region score is strongly significant (*t* = 2.642, d.f. = 45.316, *p* = 0.011, limbed mean = −1.985, SL mean = −2.507) (figure 5*e*). Limbed taxa often have scaling-independent rib shape change at axial boundaries and, in general, snake-like taxa do not.

## 4. Discussion

The maximum-likelihood models reveal consistency in both the number and pattern of regions among 56 taxa, spanning multiple independent transitions to a snake-like body form (figure 2). These models exhibit more pronounced differences between some phylogenetic groups than between different body types, as exemplified by the unique forms of teiids or acrodontans (figure 3*d*). A caveat to our results is that we were unable to calculate intraspecific variation due to sample limitations, and the potential for high variability to provide a confounding or misleading signal in our analyses cannot be discounted. However, consistency in region numbers among examined taxa and in heterogeneity differences between limbed and snake-like taxa strongly indicate that intraspecific variation is small relative to the patterns we recover across the diversity of squamate phylogeny. In contrast to the hypothesis of deregionalization, our results imply the retention of existing axial patterning, increasing the number of segments within each region for snake-like forms [61]. This aligns with existing evidence supporting the retained regionalization of the vertebral column across Squamata [22], including distantly related groups that converge on the snake-like body form [62].

The apparent homogeneity of the snake-like form arises from reduced intra-ribcage shape differences, with ancestral shape reconstruction showing further decreases in heterogeneity at the base of the boid and colubrid snake clades (figure 5*a*). These heterogeneity reductions are attributed to non-uniform shape changes along the axis, with anterior and posterior rib shapes aligning more closely with the mid-axis morphology (figure 5*b–d*). Distinct boundaries based on allometry-independent shape changes are lost, leaving regions discernible only by scaling (figure 5*d,e*). Notably, the loss of allometry-independent regional boundaries is a feature common across convergent snake-like lineages. This suggests a potential link between the developmental shape generation of ribs and size variables. As observed in human embryos [63], rib growth progresses through common morphological forms at all axial positions, but shape differences are introduced by heterochronic variation in rib development. Such heterochrony at axial boundaries might explain consistent allometric differences in regions observed in limb-reduced taxa. Mechanisms of ontogenetic allometry have been previously suggested to have a role in vertebral column evolution in certain clades of snakes [24,64]. Study of growth series in such species would determine whether this mechanism indeed drives the observed patterns in ribs. Ultimately, the macroevolutionary transition towards cryptic regionalization in snake-like lineages results from a convergence in shape towards mid-axis morphology, reducing overall axial heterogeneity.

Convergence towards a common mid-axis morphology among snake-like taxa may be attributable to selective forces as ribs transition from a role supporting pectoral appendages to providing locomotory motion through contact with substrate. In limbed taxa, the functional requirements for ribs differ according to axial position [65,66]. In response to the unique forces acting through the anterior-most ribs during locomotion, some limbed species have independently evolved dorsoventrally elongated costovertebral joints to passively dissipate forces [65,67]. These modified articular surfaces are not pervasive through limbed squamates, which is partly responsible for the high interspecies rib shape variability at the anterior of axes (figure 5*d*). In contrast to limbed taxa, functional requirements are not so discretely regionalized in snakes. All ribs at all pre-cloacal axial positions share common locomotor requirements [67], transmitting force from epaxial muscles to the body wall and the environment [68–70] and connecting to the skin via costocutaneous muscles that can propel the animal during locomotion [71]. In snake-like squamates, locomotion proceeds via similar mechanisms, using the whole axial skeleton for propulsion [72–74]. Thus, ribs along the axis of a snake-like skeleton face similar locomotor functional requirements and so have evolved a consistent morphology by modification of ribs in anterior and posterior regions, rather than a loss of regional boundaries.

Unlike functional pressures from locomotor requirements, which are more homogonous along the axis in snake-like forms than limbed lizards, snakes have unique factors that may contribute to functional regionalization in the ribs. For example, while ribs in snake-like squamates contribute to ventilation as in limbed taxa [75], the anatomy is highly adapted. The extent of the lung is variable and can extend through up to 80% of the pre-cloacal axis in snakes [76]. Similarly, the position of the heart in snakes is variable, associated with different evolutionary histories and habitat uses, and is linked to shifts in patterns of regionalization in the vertebral column [23,25,27,77]. The variable extent of lungs and heart might, therefore, contribute to some of the variation in region boundary position seen across snakes (figure 2).

While the methodology implemented here determines morphological regions that are statistically distinct, establishing homology of these detected regions across taxa requires careful consideration. While sternal articulation traditionally defines the cervicothoracic boundary [41], association between the sternal apparatus and the anterior rib region boundary is not seen across limbed squamates. Instead, a stronger association is evident between the pectoral girdle position and the posterior boundary of the anterior-most region (figures 2, 3; electronic supplementary material, 6) and is so consistent that this boundary is taken to be defined by girdle position in limbed taxa and to be homologous to the cervicothoracic boundary. However, in the absence of direct skeletal structures to homologize a boundary in snakes, alternate indicators must be considered. Absolute correspondences cannot be definitively drawn given the variability in vertebral counts seen within species of snakes [78], but when compared at resolution of subsampling procedure of 4% intervals along the length of the ribcage, we see an association between the anterior-most regional boundary and the anterior expression boundaries of *HoxC6* and *HoxB9* [11,13] (figure 4), suggesting the anterior-most region boundary represents the cervicothoracic boundary. This matches the correspondence between the anterior regional boundary resolved in this study and the boundary of *HoxC6* expression in *Anolis carolinensis* [13]. The effect of *HoxC6* in determining axial identity and rib position at this transition has been observed in homeotic transitions of cervical to thoracic identities in mutant mice [79]. The cervical expression of *HoxC6* is documented across a range of taxa [28] including *Varanus niloticus* [80], *Alligator mississippiensis* [81], *Pogona vitticeps* and *Anolis carolinensis* [13]. *HoxB9* is also known to be associated with cervicothoracic boundary patterning in determining the identity of the first rib [82]. Additionally, other features associated with the anterior rib region boundary affirm its homology to the cervicothoracic boundary of limbed forms, including the presence of muscles associated with the boundary in other squamates [43,83,84]. Comparisons of described posterior origins of primaxial-derived muscles [43] with the boundaries resolved in this analysis, MSC, MSsC and MRCA show remarkable conservation in *Pantherophis guttatus* (figure 4). The muscle origins for the latter vary more across other snake species but MSC and MSsC are within a single sampled interval of all but one snake (electronic supplementary material, 10). Consequently, while decoupled from skeletal structures, we suggest that the cervicothoracic boundary persists in the evolution of the snake-like body form, with its homology identified through association with other primaxial features, myological markers and the expression of key developmental patterning genes.

The retention of boundary patterns and their homologous regions is inconsistent with hypotheses of deregionalization in the evolution of the snake-like body form. Instead, the data support a scenario involving dissociation between primaxial and abaxial patterning [22,85]. Conserved anterior primaxial boundaries are disassociated from abaxial-derived structures, such as pectoral elements. That this uncoupling is seen across independent lineages that evolve snake-like body forms may indicate that this is a consistent feature of this evolutionary transition. It is notable that even among limbed taxa the predicted degree of interaction between abaxial and primaxial patterning, observable as a tight link between the position of sternal apparatuses and region boundaries, was not borne out in the sampled taxa (electronic supplementary material, 6). Evidence for this primaxial/abaxial dissociation is observed in the anterior shift of attachment sites for abaxial-derived pectoral girdle and hypaxial muscles in sampled snake-like taxa [43,84] (electronic supplementary material, 10). Specifically, the anterior origins of the abaxial-derived *M. transversus* and *M. obliquus internus, pars dorsalis,* considered to be indicative of the cervicothoracic boundary in squamates, are anterior to the resolved boundary in primaxial-derived ribs from *Pantherophis* and other snakes (figure 4; electronic supplementary material, 10). Hoffstetter & Gasc [41] previously noted similar shifts in features and described it as a relative dissociation of the normal cervicothoracic boundary, an observation previously construed as evidence for deregionalization. For example, posterior expansion of cervical identity was postulated [16,17] or the spread of thoracic identity anteroposteriorly throughout the axis, initially based on study of *Hox* expression boundaries [8], but later supported by the anterior observation of thoracic features such as ribs, hypaxial muscles and the pleuroperitoneal cavity [86]. However, these shifts in relative position of some features may instead represent developmental decoupling of the abaxial- and primaxial-derived tissues across the LSF [85]. Our analysis finds these trends to be common to all instances of convergent evolution of the snake-like body form.

The evolution of the superficially homogenous snake-like body form is not the result of deregionalization. Instead, employing maximum-likelihood models of segmented linear regression and geometric morphometrics reveals consistent ribcage regionalization patterns across both limbed and snake-like squamate taxa. This analysis highlights shared features of the evolutionary transition among convergent lineages, shedding light on the macroevolutionary mechanisms driving these changes. It becomes evident that adaptations in individual rib shapes, coupled with the orchestration of ribcage regionalization, allow the finely tuned development of a highly adapted body form.

## Data Availability

Raw landmark data and code can be found on Dryad [87]. The data generated through subsequent analyses are included as electronic supplementary material. Supplementary material is available online [88].
